# Biomass tar cracking and syngas production using rice husk char-supported nickel catalysts coupled with microwave heating

**DOI:** 10.1039/c8ra09045a

**Published:** 2018-12-06

**Authors:** Qing Dong, Huaju Li, Shuping Zhang, Xiangqian Li, Wa Zhong

**Affiliations:** School of Life Science and Food Engineering, Jiangsu Provincial Engineering Laboratory for Biomass Conversion and Process Integration, Huaiyin Institute of Technology Huaian 223003 China dongq@hyit.edu.cn lixiangq_hyit@sina.com +86-517-83559216 +86-517-83559216; Jiangsu Provincial Engineering Laboratory for Advanced Materials of Salt Chemical Industry, National & Local Joint Engineering Research Center for Deep Utilization Technology of Rock-salt Resource, Huaiyin Institute of Technology Huaian 223003 China; Division of New Energy Science and Engineering, School of Energy and Power Engineering, Nanjing University of Science and Technology Nanjing 210094 China

## Abstract

In the present work, biomass pyrolysis tar cracking and reforming for high quality syngas production using a rice husk char (RHC)-supported nickel catalyst (Ni/RHC) coupled with microwave heating was investigated. The Ni/RHC catalyst exhibited a high catalytic performance on tar removal and contributed well to the production of CO and H_2_. The conversion efficiency could reach up to 97.3%, and the CO and H_2_ yields were 274.0 ml g^−1^ and 248.9 ml g^−1^, respectively, at 700 °C, under microwave conditions, when the nickel loading amount was 10.42 wt% of the support. The tar conversion efficiencies and syngas yields significantly increased as the cracking temperatures increased from 500 °C to 700 °C and the nickel loading amount increased from 0 to 10.42 wt%. The Ni/RHC catalysts became more effective for tar removal and the production of syngas increased under microwave conditions compared to the results obtained under conventional conditions.

## Abstract 2

We explored the effect of Ni/RHC catalyst on tar removal and syngas production under microwave conditions.

## Graphical abstract

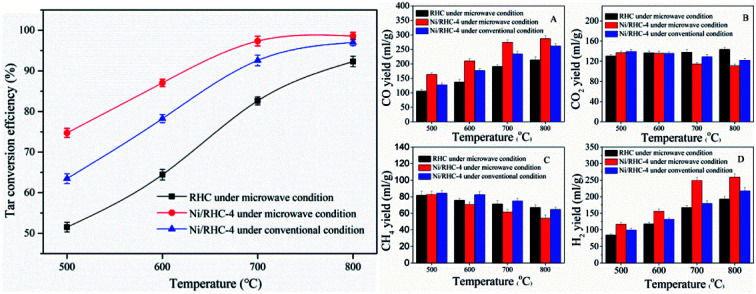

## 1. Introduction

Syngas (H_2_ + CO) is a major starting material for the production of a sequence of platform chemicals and synthetic fuels.^1^ It can be generated from many sources, including natural gas, coal, biomass and so on. Biomass is acknowledged as a potential source for synthesis gas production because of its abundance, renewability and cleanness.^2^ Gasification/pyrolysis is recognized as one of the most effective technologies applied to convert biomass cleanly into syngas. However, biomass tars are normally generated along with the target product during the gasification or pyrolysis process. Biomass tar needs to be removed to avoid damaging and clogging downstream pipes or equipment.^3^ Consequently, it is important to achieve efficient tar removal for the clean syngas production from biomass gasification or pyrolysis.

At present, several technologies, including the physical method,^4^ thermochemical cracking,^5^ and catalytic cracking,^6^ have been extensively tested for biomass tar removal. Among these technologies, catalytic cracking is accepted as the technology with the most potential for commercial applications because of its high reaction rate, relatively mild reaction conditions and production of additional syngas.^7^ Currently, the application of various types of catalysts, including natural minerals,^8^ carbon-based catalysts,^9^ and metallic catalysts, such as nickel-based catalysts,^10^ and iron-based catalysts,^11^ has been reported in the published literature. Among the above-mentioned catalysts, carbon-based catalysts, employed for the biomass tar cracking/reforming, are becoming the spotlight in the biomass gasification and pyrolysis fields. Carbon materials, obtained from biomass pyrolysis/gasification, have already been proved to exert a positive influence on biomass tar removal because of their developed pore structure and abundant surface oxygen-containing functional groups.^12^ Moreover, these carbon materials have a low cost, and can be simply regenerated after deactivation since part of the coke on the catalysts can directly be gasified during the tar removal.^13^ However, the catalytic activity of carbon materials exclusively is unsatisfactory compared to some metallic catalysts.^14^ In relation to the catalytic activity and economy, nickel-based catalysts were acknowledged as the most potential for biomass tar cracking/reforming for syngas production. Natural minerals and metal oxides are generally applied as the support for the nickel-based catalysts.^10,15^ It is unfortunate that these supports have a relatively high cost and are easily deactivated due to the generation of carbon deposition.^3^ In recent years, some scientists demonstrated that biochar-supported nickel catalysts could get over the above-mentioned disadvantages and exert a remarkable effect on the cracking of biomass tars into syngas.^7,16^ However, all these studies were carried out under conventional electrical heating condition, where relatively high reaction temperatures (800–900 °C) were generally required for the efficient tar removal, which inevitably results in an adverse cracking condition and more energy consumption.

In a comparison to the conventional heating methods, microwaves, acting as an internal heat source, can directly heat the target materials without heating the entire furnace, which saves time and energy.^17^ Moreover, microwave heating has been demonstrated to be much favorable to some heterogeneous catalytic reactions.^18^ More and more researches have been conducted on tar cracking and reforming using microwave heating.^19–22^ The obtained results showed that the tar conversion and syngas production were more efficient under microwave condition than under conventional condition when the other reaction conditions were similar. Previous work reported the influence of various biochar-supported metallic catalysts on the biomass pyrolysis.^23^ The results indicated that the biochar-supported nickel catalyst promoted the gas production mainly at the expense of the liquid products. However, it is unfortunate that the liquid yields were still maintained at a high level (about 12 wt%). The effect of the heating method on the catalyst performance was also not involved.

To the best of our knowledge, no researches were performed on the employing of the Ni/RHC catalysts for biomass tar removal coupled with microwave heating. In the present work, *ex situ* biomass pyrolysis tar cracking and reforming over the Ni/RHC catalyst was investigated under microwave condition using a two-stage fixed bed reactor. The influences of reaction temperature and Ni loading amount on biomass tar removal combined with syngas production were described in detail. The catalytic cracking of tar was also carried out under electric heating condition to demonstrate the influence of heating method on tar removal and syngas production.

## 2. Experimental section

### 2.1 Materials

The rice husk, harvested from Huaian city, China, was selected as the feedstock in the present experiments. The shattered rice husk was sieved and the particles sizes ranging from 20 mesh to 40 mesh were used for the present study. The chosen materials were then dried for 12 h at 105 °C prior to the tests. Proximate analysis was conducted on the basis of the American Society of Testing Materials (ASTM) procedures. The ultimate analysis was conducted using a Vario EL-III elementar (ELEMENTAR Analysensysteme GmbH). The obtained results are indicated in Table 1.

**Table 1:** Proximate and ultimate analyses of RHC (dry basis, wt%)

Proximate analysis			Ultimate analysis				
Volatile matter	Fixed carbon	Ash	C	H	N	O^a^	S
64.6	13.02	22.38	40.64	6.1	0.35	30.43	0.1

a Calculated by difference.

### 2.2 Catalyst preparation

The same rice husk was applied for the production of the RHC in this work. The particles, with the sizes of 100–200 mesh, were pyrolyzed in a microwave reactor. The detailed description of the experimental setup has been presented in the previous work.^23^ The rice husk pyrolysis was carried out under microwave condition. 100 l h^−1^ of nitrogen was used as the protection gas for the experiment. The rice husk was heated for 15 min at 900 W. The obtained RHC was cooled down to room temperature in the nitrogen atmosphere. The catalysts were prepared *via* the incipient wetness method. About 20 g of the obtained RHC was then impregnated in the nickel nitrate solution, with the following amounts of nickel nitrate: 0.01 mol, 0.02 mol, 0.04 mol and 0.06 mol, respectively. Then, the RHCs, containing different amounts of nickel nitrate, were dried at 105 °C for 12 h. The dried RHCs were then heated in the above-mentioned microwave reactor at 800 W in the nitrogen atmosphere for 10 min. The obtained Ni/RHC catalysts were used for the present work. The catalysts were noted as Ni/RHC-1, Ni/RHC-2, Ni/RHC-4 and Ni/RHC-6, according to the addition amount of nickel added, respectively.

The textural properties of the catalysts were analyzed by applying a Micromeritics instrument ASAP 2020. The nitrogen adsorption method at 77 K was employed for the analysis. Brunauer–Emmett–Teller (BET) model was applied to determine the specific surface area. The total pore volume was calculated *via* the amount of nitrogen adsorbed at a relative pressure of *p*/*p*_0_ = 0.98. The amounts of the Ni and other metal element present in the RHC were determined applying inductively coupled plasma-optical emission spectra (ICP-OES). Details of the procedure of the ICP-OES analysis have been described in a published report.^24^ The contents of the oxygen-containing functional groups on the fresh and used catalysts were analyzed using Boehm titration method.^25^ The Ni/RHC catalysts, before and after reactions, were also analyzed with the help of a D/max 2500VL/PC X-ray diffractometer (XRD) with Cu Ka radiation (40 kV, 200 mA) from 10° to 80° with a step of 0.02° s^−1^.

The quantity of the coke deposited on the catalyst was estimated with the help of a Perkin Elmer Pyris-1 thermogravimetric analyzer (TGA). Prior to the TG analysis, the fresh and used catalysts were heated at 500 °C under N_2_ and H_2_ atmosphere at 200 ml min^−1^ (H_2_/N_2_ = 1 : 9) in the tar cracking reactor to eliminate the effect of the catalyst weight changes generated from the formation of NiO. The reduced catalysts by H_2_ were then subjected to the TG analysis. About 10 mg of the catalysts was employed in each experiment. The catalysts were heated from room temperature to 900 °C at 10 °C min^−1^ under air atmosphere, and then held for about 5 min after reaching the desired temperature.

### 2.3 Experimental apparatus and procedure

**Fig. 1:**
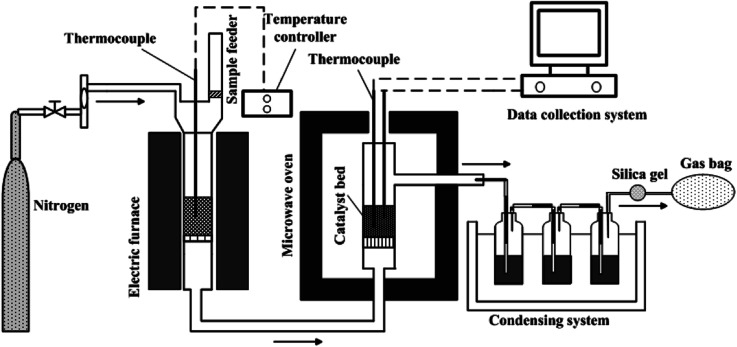
Schematic diagram of the experimental setup for microwave-assisted tar cracking.

Fig. 1 displays the schematic diagram of microwave-assisted tar cracking and reforming. The whole experimental system was made up mainly of an electric furnace and a microwave oven. Details of the electric furnace can be found in previous work.^9^ The biomass pyrolysis was conducted at 600 °C in the electric furnace. About 10 g of the rice husk was put in a quartz tube (60 cm height, 3.8 cm inner diameter) contained in the electric furnace as soon as the temperature of the catalyst bed in microwave oven reached the target temperature, and held there for 30 min. Nitrogen at a flow rate of 200 ml min^−1^, was applied as the protection gas during the experiments. The reactor outlet in the electric furnace was connected to the inlet of the cracking reactor in microwave oven with the help of quartz tubes which was maintained at 300 °C by a heater band in order to avoid the condensation of the pyrolysis volatiles. The microwave oven for tar catalytic cracking in the present work was converted from the one mentioned in the published report.^23^ The catalytic cracking/reforming of the tar vapors was performed in the microwave oven, with the maximal power of 2 kW at 2.45 GHz. About 5 g of the catalysts were put into a quartz reactor (50 cm height, 3.8 cm inner diameter) and the height of the catalyst bed was about 4 cm. The catalyst bed temperatures were set as 500 °C, 600 °C, 700 °C and 800 °C. Two K-type thermocouples were applied for measuring the catalyst bed temperatures. The average values of the temperatures measured by these two K-type thermocouples were adopted in this work.

The catalytic cracking and reforming of the tars under conventional electric heating condition were carried out using the same electric furnace mentioned above. The operating parameters were set as the same as those under the microwave condition in order to make a comparison between the microwave and conventional condition. The experiment of the RHC pyrolysis without catalytic cracking was also conducted to reveal the catalyst performance. The pyrolysis vapors, after catalytic cracking and reforming, were passed through the condensate system which was composed of three ethanol-containing flasks immerged in an ice-water bath. The pyrolysis vapors, without suffering catalytic cracking and reforming, were passed through the same condensate system. The tar residues on the tube were washed by ethanol. The other tars were collected from the ethanol-washing bottles. The tars from the tube and washing bottles were mixed. Subsequently, the mixture was subjected to evaporation, in order to remove the solvent, at 60 °C. The weight of the remnant liquid products, defined as tar in the present study, was determined on the basis of the weight difference between the empty flask and the flask after ethanol evaporation. Then, the liquid fraction yield can be obtained on the basis of the biomass weight. The residue char in the electric oven was weighed, and its yield was also determined on the basis of the biomass weight. Then, the gas yield can be determined by mass balance. The tar conversion was be calculated by eqn (1):1
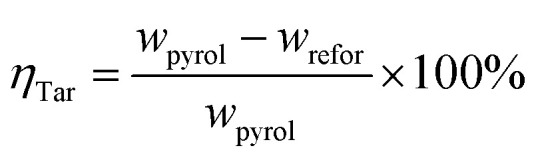
where *η*_Tar_ is the tar conversion efficiency (%); *w*_pyrol_ represents the tar yield without catalytic cracking (g g^−1^); and *w*_refor_ is the tar yield after catalytic cracking (g g^−1^). The permanent gaseous products were collected in the gas bags for GC analysis. The yield of each gas composition can be calculated by the following equations:

2 *w*_H_2__ = *V*_m_ × *x*_H_2__ × *w*_gas_/*M*_ave_ × 100%

3 *w*_CO_ = *V*_m_ × *x*_CO_ × *w*_gas_/*M*_ave_ × 100%

4 *w*_CO_2__ = *V*_m_ × *x*_CO_2__ × *w*_gas_/*M*_ave_ × 100%

5 *w*_CH_4__ = *V*_m_ × *x*_CH_4__ × *w*_gas_/*M*_ave_ × 100%

where *w*_H_2__, *w*_CO_, *w*_CO_2__ and *w*_CH_4__ are the yields of H_2_, CO, CO_2_ and CH_4_ (ml g^−1^), respectively; *x*_H_2__, *x*_CO_, *x*_CO_2__ and *x*_CH_4__ are the volumetric concentrations of H_2_, CO, CO_2_ and CH_4_ (N_2_ free, %), respectively; *w*_gas_ represents the gas yields based on the biomass (g g^−1^); *M*_ave_ is the average molar mass of the gaseous products (g mol^−1^), which can be calculated based on the volumetric concentrations and molar mass of the gaseous compounds; and *V*_m_ is the standard molar volume (224 ml mol^−1^).

The experiments were repeated three times to verify the obtained values.

### 2.4 Products analysis

The chemical compositions of the residual tar in the flask after ethanol evaporation were determined by a gas chromatography-mass spectrometry (GC/MS; Agilent 7890A-5975C) equipped with a Varian Cp-sil 8cb capillary column (30 m length × 0.25 mm ID diameter × 0.25 μm film thickness). The details of the operating parameters can be found in the published report by Dong *et al.*^9^

The compositions of the obtained permanent gases were determined *via* a 6890N gas chromatography with a dual thermal conductivity detector (TCD). A molecular sieve 5A and GDX401 were used as the chromatographic columns. High-purity argon was employed as the carrier gas at a 15 ml min^−1^ flow rate. The temperatures of the detector, oven and injector were set as 150, 80, and 100 °C, respectively.

## 3. Results and discussion

### 3.1 Catalyst characterization

The textural properties of the fresh and used catalysts are presented in Table 2. It is found that the RHC has a relatively high value of the BET surface area (60 m^2^ g^−1^). This value is much higher than that of the RHC obtained from conventional pyrolysis (0.44–17 m^2^ g^−1^).^3,26^ This suggested that microwave heating had a positive influence on the textural properties of RHC. The reason could probably be that the pyrolysis volatiles could be rapidly released under microwave heating, which avoided the deposit of the cracking carbon on the pores. The addition of nickel into the RHC increased the BET surface area of the catalysts which could be resulted from the preparation methods and activation of precursors under microwave condition.^23^ The maximal BET surface area of 200 m^2^ g^−1^ was obtained for Ni/RHC-4 catalyst. When the loading amount was further increased, the BET surface area showed an adverse decreasing tendency, from 201 m^2^ g^−1^ for Ni/RHC-4 to 156 m^2^ g^−1^ for Ni/RHC-6. This could be probably because that more pores of the catalysts were blocked by too many nickel loadings. After reaction, the *S*_BET_ of the Ni/RHC-4 catalyst was decreased from 201 m^2^ g^−1^ to 182 m^2^ g^−1^, probably because of the that coke was formed during the reaction. However, the total pore volume was increased from 0.148 cm^3^ g^−1^ to 0.165 cm^3^ g^−1^. This could be resulted from the decomposition of the oxygen-containing functional groups and the gasification reactions which carbon took part in.

**Table 2:** Textural properties of the fresh and the used catalysts

Catalysts	*S*_BET_ (m^2^ g^−1^)	Total pore volume (cm^3^ g^−1^)	Average pore diameter (nm)
RHC	60	0.032	2.15
Ni/RHC-1	126	0.070	2.22
Ni/RHC-2	178	0.123	2.76
Ni/RHC-4	201	0.148	2.95
Ni/RHC-6	156	0.133	3.40
Used Ni/RHC-4 under microwaves	182	0.165	3.59

The results of the relative contents of the metallic species in the catalyst samples are shown in Table 3. It can be seen that many types of metallic elements contained in RHC, were detected *via* ICP-OES, including K, Ca, Na, Mg and Fe, which were all helpful for the tar cracking.^27,28^ In addition, the effective loading amounts of nickel on the RHC were 2.53 wt%, 5.21 wt%, 10.42 wt% and 16.86 wt% for Ni/RHC-1, Ni/RHC-2, Ni/RHC-4 and Ni/RHC-6, respectively,which were all slightly lower than the theoretical values. H^+^ can be formed when Ni(NO_3_)_2_ dissolves in water. Parts of the metal element can dissolve in water and it is inevitable that the loss of parts of the metal element took place. In addition, the loading of Ni into the support would increase the mass of the catalyst, which would also decrease the content of metallic species contained in the RHC support.

**Table 3:** Relative content of metallic species in catalyst samples (mg kg^−1^, dry basis)

Catalysts	K	Ca	Na	Mg	Fe	Ni
RHC	2568	782	60.5	345	89.2	—
Ni/RHC-1	1965	693	58.3	302	68.5	25 323
Ni/RHC-2	1233	586	<50	282	72.3	52 158
Ni/RHC-4	864	537	<50	167	63.4	104 210
Ni/RHC-6	572	325	<50	139	<50	168 561

The Boehm titration method,^25^ can be applied to determine the oxygen-containing functional groups on the RHC surface, including –OH, –COOH and C=O. Table 4 showed the contents of these functional groups, before and after catalytic cracking at 700 °C. It can be found that the contents of –OH, –COOH and C=O groups were all lowered after the catalytic cracking. This indicated that parts of these functional groups participated in the cracking and reforming reaction. It was worth noting that the contents of the three types of functional groups were all lower for the used RHC and Ni/RHC-4 catalysts under microwave heating than those under conventional heating, suggesting that microwave heating was probably favorable to the reactions between the RHC and biomass tars. The loading of Ni decreased the contents of oxygen-containing functional groups. This can be explained by the two following points: (1) the loading of Ni onto the support surface would decrease the quantity of these functional groups on the support surface; and (2) parts of these functional groups probably decomposed when the support was heated during the catalyst preparation. In a comparison to the contents of –OH, –COOH and C=O in the fresh RHC, those in the used RHC catalyst under microwave condition were decreased by 51.49%, 59.57% and 32.39%, respectively. Compared to the contents of –OH, –COOH and C=O in the fresh Ni/RHC-4, those in the used Ni/RHC-4 under microwave condition were decreased by 68.79%, 62.5% and 44.44%, respectively. Therefore, it can be concluded that the loading of Ni could promote the decomposition of these functional groups during microwave catalytic cracking.

**Table 4:** Boehm titration results of the fresh and used catalysts (mmol g^−1^)

Catalysts	–OH	–COOH	C=O
Fresh RHC	0.202	0.047	0.071
Used RHC under microwave condition	0.098	0.019	0.048
Used RHC under conventional condition	0.134	0.029	0.059
Fresh Ni/RHC-4	0.157	0.032	0.063
Used Ni/RHC-4 under microwave condition	0.049	0.012	0.035
Used Ni/RHC-4 under conventional condition	0.065	0.020	0.042

**Fig. 2:**
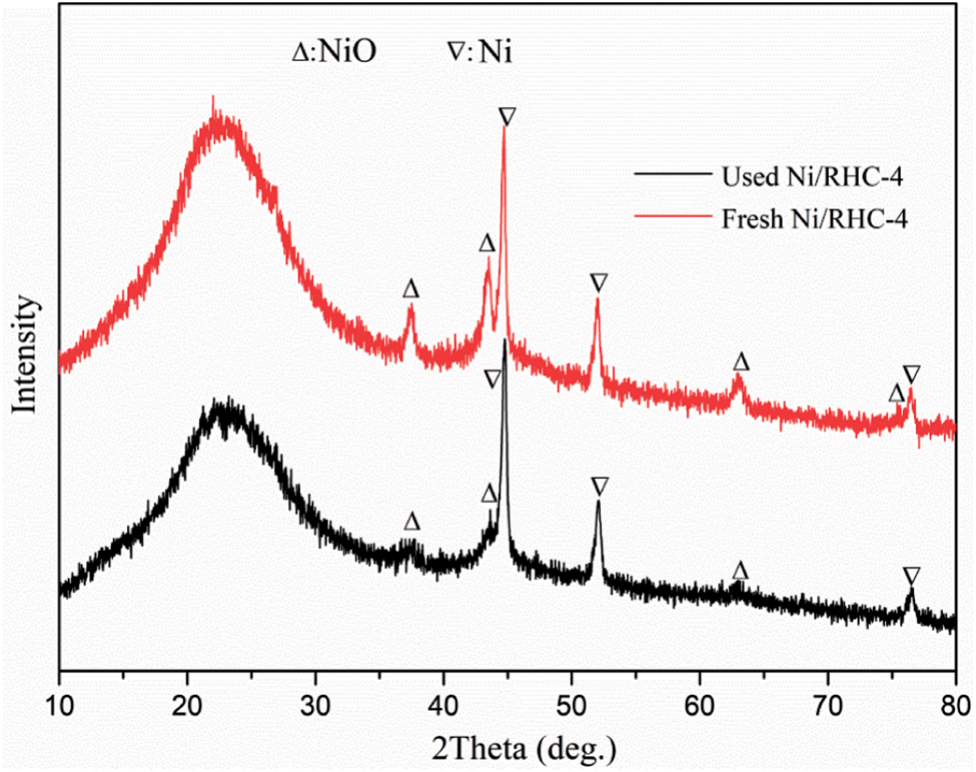
XRD patterns of the fresh and used Ni/RHC-4 catalysts.

The crystal phases of the fresh and spent Ni/RHC-4 catalysts for microwave catalytic cracking at 700 °C were analyzed using XRD and the result is shown in Fig. 2.

As shown in Fig. 2, the catalysts showed a broad characteristic peak around 2*θ* = 22.5°, representing the crystal structure of amorphous silica.^3^ For the fresh catalyst, the diffraction peaks representing nickel oxide were found at 2*θ* = 37.29°, 43.63°, 63.00° and 75.37° (PDF # = 22-1189), and the diffraction peaks, representing metallic nickel (Ni^0^) were observed at 44.77°, 52.10° and 76.48° (PDF # = 65-2865). During the catalytic cracking process, parts of the nickel oxide in the fresh catalyst might be reduced to metallic nickel at the presence of carbon, H_2_ and CO,^29^ which resulted in the decreases in the height of the diffraction peaks of nickel oxide at 2*θ* = 37.29°, 43.63° and 63.00° and the disappearance of the nickel oxide diffraction peaks at 2*θ* = 75.37°.

### 3.2 Tar conversion

**Fig. 3:**
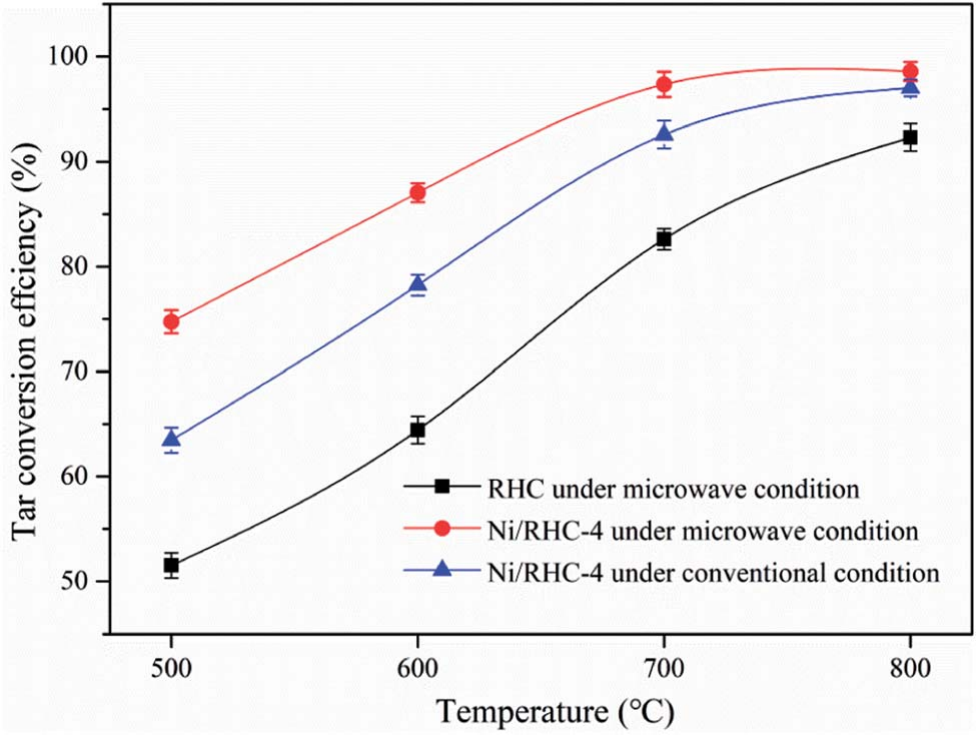
Tar conversion efficiency at different cracking temperatures and under different heating methods.

The influences of the cracking temperatures and heating methods on tar conversion were studied at the temperatures ranging from 500 °C to 800 °C. It can be found from Fig. 3 that the temperature increase was favorable to the tar conversion. The tar conversion efficiencies were increased from 51.5% to 92.3% for the RHC catalyst under microwave condition, from 74.7% to 98.6% for the Ni/RHC-4 under microwave condition and from 63.5% to 97.0% for the Ni/RHC-4 under conventional condition, respectively. Temperature is one of the key parameters affecting biomass tar conversion. The high temperature was favorable to the tar cracking reactions which are highly endothermic.^30^ However, too high cracking temperatures above 700 °C were not recommended under microwave condition in this study because the conversion efficiency was only increased by 1.2% as the temperature was enhanced to 800 °C.

The type of catalyst is also one of the important parameter influencing the tar removal. As shown Fig. 3, the tar conversion efficiencies for the Ni/RHC-4 catalysts, regardless of the heating method employed, were always higher than those for RHC. This suggested that nickel could exert a significant positive influence on tar removal. It was reported that the biochar from biomass pyrolysis/gasification is a reasonable catalyst for the efficient cracking of tars because of its developed pore structure, high adsorption capability to tars and excellent surface properties containing some metallic species that showed a positive effect on tar removal.^3^ However, higher temperatures (≥800 °C) were generally required for the tar efficient cracking over the pure RHC catalyst.^3,31^ The tar removal efficiency could be significantly promoted by the loading of nickel into the RHC. It has been demonstrated that Ni^0^ possesses high reforming activity of hydrocarbon *via* the activation of the C–H and C–C bonds in hydrocarbon molecules on the metal nickel surfaces.^7^ It is especially pointed out that the RHC, as a catalyst support, could contribute well to the utilization of the high catalytic performance of nickel.^16^ The developed pore structure was favorable for the adsorption of tars on the active sites. Moreover, the nickel oxide can be reduced to Ni^0^ by the carbon contained in RHC. Therefore, the Ni/RHC catalyst showed a higher catalytic activity for tar conversion than the pure RHC.

The tar conversion efficiencies for the Ni/RHC-4 catalysts subjected to microwave heating were all higher than those under conventional condition at the same temperatures. Compared to conventional heating, the tar conversion efficiencies were increased by 17.8%, 11.3%, 5.1% and 1.6% under microwave condition at 500 °C, 600 °C, 700 °C and 800 °C, respectively. However, the difference in the tar removal efficiency was decreased as the cracking temperature increased, suggesting that microwave heating could be more effective on tar removal than conventional heating at lower temperatures. Similar results can be found in published reports, in which the difference between the microwave and conventional pyrolysis of microalgae was also found to be decreased as the pyrolysis temperature increased, in terms of the product yields. During the conventional heating process, the heat flows passed through the surfaces into the inside of the samples. In contrast, microwaves can directly generate heat within the catalyst due to its interaction with the samples, which is bound to result in the generation of the higher temperatures in the interior of the samples. In addition, during microwave heating, a lot of small hotspots have been discovered when carbon materials were heated by microwaves.^32^ Whereas the overall temperature corresponds to those measured by the thermocouples, the temperatures in these “hot spots” must be much higher. Therefore, the presence of the hotspots can promote the heterogeneous reactions between the solid catalyst and gases.^32^ In other words, it is the pseudo-catalytic effect of microwave heating.^33,34^ It is worthwhile noting that the tar conversion efficiency reached up to 97.3% at 700 °C and was only slightly increased as the temperature increasing to 800 °C, for the Ni/RHC-4 catalyst under microwave condition. The tar conversion efficiency at 700 °C under microwave condition was even higher than that at 800 °C under conventional condition, also indicating that microwave heating was more effective on heterogeneous catalytic reactions.

**Fig. 4:**
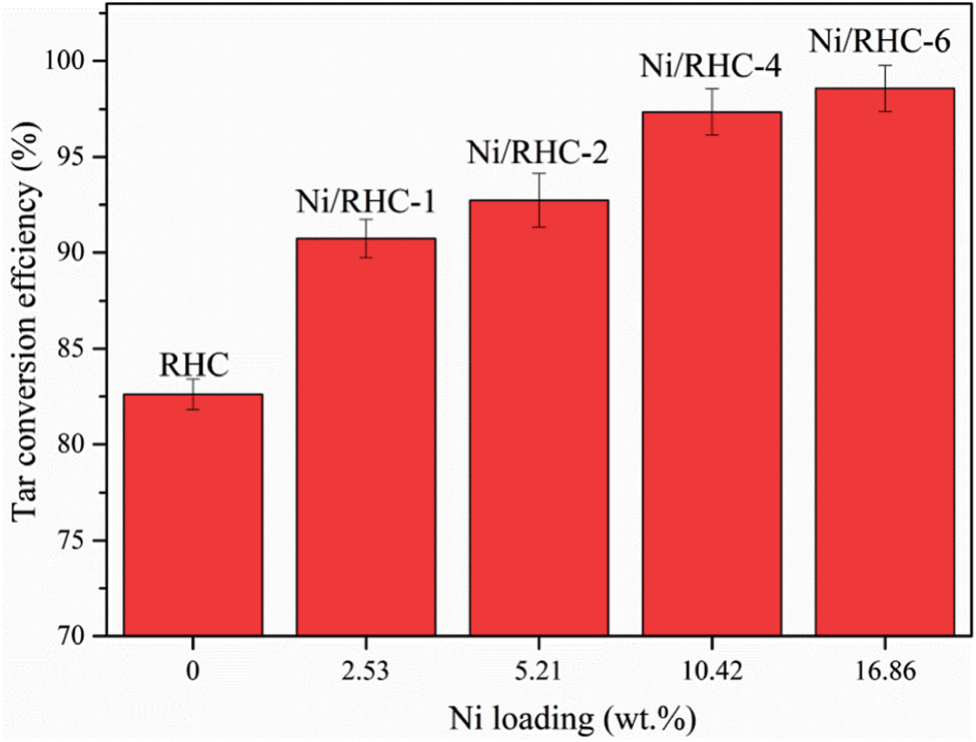
Dependence of the tar conversion efficiency on the nickel loading at 700 °C under microwave condition.

The dependence of the tar conversion efficiency on the nickel loading amount at 700 °C is presented in Fig. 4. The tar conversion efficiencies were increased from 78.6% to 98.6% with the loading amount increasing from 0 wt% to 16.86 wt%, indicating that the loading amount had a significant effect in the tar cracking. This could be probably because that more Ni^0^ was produced during tar cracking when the content of nickel element loaded into the support was higher.^7^ A rapid increase in tar removal was found as the Ni loading amount increased from 5.21 wt% to 10.42 wt%. However, the tar conversion efficiency showed a non-significant increase as the loading amount was further enhanced from 10.42 wt% to 16.86 wt%. A nickel loading amount that was too high decreased the surface area of the catalyst (Table 2). Therefore, the catalyst performance could be deteriorated by too much nickel loading.

### 3.3 Gaseous products

**Fig. 5:**
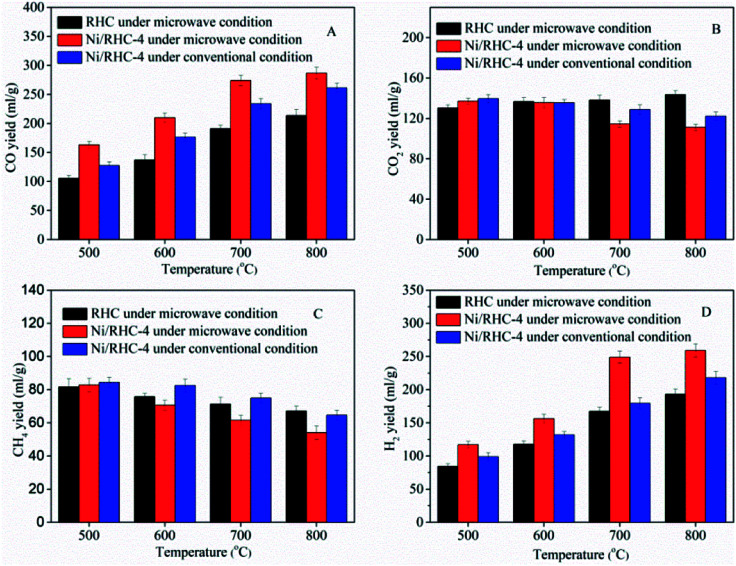
Yields of the gas compositions at different cracking temperatures.

The non-condensable gaseous products consisted mainly of CO, CO_2_, H_2_ and CH_4_. The yield of each composition, depending on the cracking temperatures, is presented in Fig. 5. The yields of CO and H_2_ were both enhanced as the temperature increased from 500 °C to 800 °C in all operating situations, indicating that higher cracking temperatures were favorable to the production of syngas. The CO yields were increased from 105.4 ml g^−1^ to 213.5 ml g^−1^ for the RHC catalyst under microwave condition, from 163.2 ml g^−1^ to 286.6 ml g^−1^ for the Ni/RHC-4 under microwave condition and from 127.3 ml g^−1^ to 261.3 ml g^−1^ for Ni/RHC-4 under conventional condition. The H_2_ yields were increased from 84.3 ml g^−1^ to 192.9 ml g^−1^ for the RHC catalyst under microwave condition, 116.9 ml g^−1^ to 259.0 ml g^−1^ for Ni/RHC-4 under microwave condition and 98.9 ml g^−1^ to 217.8 ml g^−1^ for the Ni/RHC-4 under conventional condition. The volumetric concentration of the syngas reached up to 74.82% when the Ni/RHC-4 catalyst was employed at 700 °C under microwave condition. The tar cracking and reforming mechanisms could be generally expressed by several heterogeneous and homogeneous reactions (reactions (6)–(13)).^13^

6 Tars → C + C_*n*_H_*m*_ + gases

7 C_*n*_H_*m*_ + *n*CO_2_ → 2*n*CO + (*m*/2)H_2_

8 C_*n*_H_*m*_ + *n*H_2_O → *n*CO + (*n* + *m*/2)H_2_

9 C_*n*_H_*m*_ + 2H_2_O ↔ *n*CO_2_ + (2*n* + *m*/2)H_2_

10 CH_4_ + H_2_O ↔ CO + 3H_2_, Δ*H*_298 K_ = +206 kJ mol^−1^

11 CH_4_ + CO_2_ ↔ 2CO + 2H_2_, Δ*H*_298 K_ = +247 kJ mol^−1^

12 C + H_2_O ↔ CO + H_2_, Δ*H*_298 K_ = +131 kJ mol^−1^

13 C + 2H_2_O ↔ CO + 2H_2_, Δ*H*_298 K_ = +173 kJ mol^−1^

On the basis of the Le Chatelier's principle, higher temperatures could promote the endothermic reactions. Therefore, it is probable that the increase of the cracking temperature could promote the reactions (eqn (6)–(13)) proceeding towards the right, which resulted in the efficient tar removal and the increase in the production of CO and H_2_. It is especially pointed out that the yields of CO and H_2_ were only increased by 4.5% and 4.1%, respectively, under microwave condition for the Ni/RHC-4 catalyst with the temperature increasing to 800 °C, indicating that a temperature above 700 °C was not worthwhile.

In a comparison to the RHC, the Ni/RHC catalyst increased the yields of CO and H_2_ significantly, regardless the heating methods employed, which was consistent with the results from the published reports.^16,26^ It was reported that the presence of Ni^0^ could play an important role in some reforming reactions (eqn (7)–(11)) in which hydrocarbons as reactants took part.^7^ However, the effect of the RHC support was non-negligible because of its good adsorption performance to these hydrocarbons, which increased the contact of the reactants and the metal active components.^13^

Compared to the conventional heating method, microwave heating showed an obvious advantage in the production of gaseous compounds. The yields of CO and H_2_ were always higher under microwave condition than those under conventional condition at the same cracking temperatures. This could be attributed to the following two factors: (a) the presence of the hot spots and micro-plasmas generated by the interaction of microwaves with the RHC supports was favorable to some heterogeneous reforming reactions (eqn (12) and (13)); and (b) parts of the carboxyl and carbonyl groups on the RHC surface were decomposed during the cracking process. The main products of the carboxyl and carbonyl group decompositions were CO_2_ and CO, respectively.^35^ The production of CO_2_ could promote the reactions (eqn (7), (11) and (13)) proceeding towards the right, which would increase the production of H_2_ and CO. The amount of these functional groups subjected to decomposition under microwave condition was higher than that under conventional condition.

**Fig. 6:**
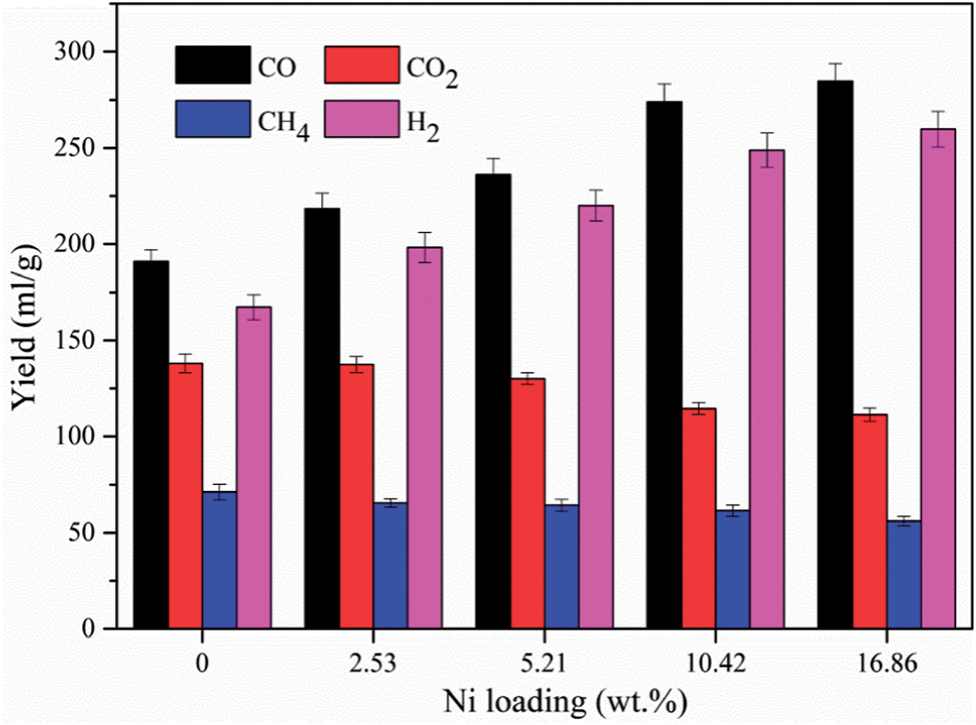
Yields of gas compositions for different Ni loading amount at 700 °C under microwave condition.

The influence of the nickel loading amount on the yields of the gas compositions was also investigated at 700 °C, and the result is presented in Fig. 6. The CO and H_2_ yields were increased from 190.9 ml g^−1^ to 284.5 ml g^−1^ and from 167.3 ml g^−1^ to 259.8 ml g^−1^, respectively, as the loading amount increased from 0 wt% to 16.86 wt%, suggesting that the loading amount could exert a significant influence on the syngas production. The rapid increases in CO and H_2_ yields were found when the Ni loading amount increased from 5.21 wt% to 10.42 wt%. The CO and H_2_ yields were merely increased 3.8% and 4.4%, respectively, when the loading amount was further increased from 10.42 wt% to 16.86 wt%. This could be resulted from that too much nickel loading destroyed the pore properties of the support, which could deteriorate the performance of the catalyst.

### 3.4 GC-MS analysis of tar

The organic phases of the tar after the catalytic cracking at 700 °C were determined using GC-MS. The identified compositions in tars consist mainly of phenol, benzene, naphthalene, anthracene, phenanthrene and their derivates. The components of the tars were classified into four groups: one-ring aromatics, two-ring aromatics, three-ring aromatics and linear-chain compounds. Phenols, including phenol, 2-methyl-phenol, 3-methyl-phenol, benzene, benzofuran and 1-ethyl-2-methyl-benzene, were the main composition of one-ring aromatics. Two-ring aromatics were mainly composed of naphthalene compounds, including naphthalene, 2-methyl-naphthalene, 1-methyl-naphthalene and 2-ethenyl-naphthalene. Anthracenes and phenanthrenes, including anthracene, 2-methyl-anthracene, 1-methyl-anthracene, phenanthrene, 1-methyl-phenanthrene and 2-methyl-phenanthrene, were mainly responsible for the three-ring aromatics. The linear-chain compounds mainly comprised acids and carbonylic compounds. The relative contents of the tar fraction obtained from different catalysts under microwave and conventional condition at 700 °C, were presented in Table 5.

**Table 5:** Relative contents of the tar fraction obtained from different catalysts under microwave and conventional condition at 700 °C

Tar fraction	Formula	Relative content (area%)			
		No catalyst	RHC under microwave condition	Ni/RHC-4 under conventional condition	Ni/RHC-4 under microwave condition
**Linear-chain compounds**					
Acetic acid	C_2_H_4_O_2_	52.32	8.74	0	0
Propanoic acid	C_3_H_6_O_2_	3.65	0.12	0	0
1-Hydroxy-2-butanone	C_3_H_6_O_2_	5.37	1.28	0	0
1,1-Diethoxy-pentane	C_9_H_20_O_2_	0.69	0.06	0	0
**Total**		**62.03**	**10.02**	**0**	**0**

**One-ring aromatics**					
Phenol	C_6_H_6_O	3.52	8.38	19.35	26.72
2-Methyl-phenol	C_7_H_8_O	6.35	5.42	2.31	0
3-Methyl-phenol	C_7_H_8_O	3.41	6.27	4.42	3.93
2,3-Dimethyl-phenol	C_8_H_10_O	0.39	0	0	0
2,4-Dimethyl-phenol	C_8_H_10_O	1.26	0.07	0	0
2-Methoxy-4-methyl-phenol	C_8_H_10_O_2_	1.05	0	0	0
4-Ethyl-2-methoxy-phenol	C_9_H_12_O_2_	0.07	0	0	0
2,6-Dimethoxy-4-(2-propenyl)-phenol	C_11_H_14_O_3_	1.13	0	0	0
Benzene	C_6_H_6_	0.86	8.54	12.76	18.25
Benzofuran	C_8_H_6_O	0.87	5.27	8.39	9.37
2-Propenyl-benzene	C_9_H_10_	0.56	0	7.40	1.41
1-Ethyl-2-methyl-benzene	C_9_H_12_	0.72	0	0	0
1-Ethyl-3-methyl-benzene	C_9_H_12_	5.96	2.61	0	0
**Total**		**26.15**	**36.56**	**54.63**	**59.68**

**Two-ring aromatics**					
Naphthalene	C_10_H_8_	3.14	15.69	25.07	30.73
2-Methyl-naphthalene	C_11_H_10_	2.56	5.72	2.32	1.56
1-Methyl-naphthalene	C_11_H_10_	1.73	4.26	3.14	2.98
2-Ethenyl-naphthalene	C_12_H_10_	2.25	0.78	0	0
1-Ethyl-naphthalene	C_12_H_12_	1.02	1.46	1.95	2.47
1,4-Dimethyl-naphthalene	C_12_H_12_	0.73	0.21	0	0
1,3-Dimethyl-naphthalene	C_12_H_12_	0.39	0.26	0	0
**Total**		**11.82**	**28.38**	**32.48**	**37.74**

**Three-ring aromatics**					
Anthracene	C_14_H_10_	0	3.64	2.23	1.58
1-Methyl-anthracene	C_15_H_12_	0	8.49	3.2	0
2-Methyl-nthracene	C_15_H_12_	0	6.55	0.89	0
Phenanthrene	C_14_H_10_	0	0.52	4.72	0.92
1-Methyl-phenanthrene	C_15_H_12_	0	3.25	0	0
2-Methyl-phenanthrene	C_15_H_12_	0	2.41	1.85	0.08
**Total**		**0**	**24.86**	**12.89**	**2.58**

The relative content of the linear-chain compounds in the tar from thermal pyrolysis without catalytic cracking reached up to 62.03%. The content of the linear-chain compounds in tars were significantly decreased by the RHC catalyst, and were completed removed when the Ni/RHC-4 catalysts were applied for the tar cracking and reforming. After the loading of nickel in the RHC, the total relative contents of one-ring and two-ring aromatics were both significantly increased, while those for three-ring aromatics showed an opposite trend. This indicated that the loading of nickel promoted the cracking of parts of three-ring aromatics into lighter compounds, including anthracene, 1-methyl-anthracene, 2-methyl-anthracene, 1-methyl-phenanthrene and 2-methyl-phenanthrene. Compared to the conventional method, microwave-assisted catalytic cracking decreased the content of three-ring aromatics by about 80.0%, indicating that microwave heating was more effective on the cracking of some heavy molecular compounds.

### 3.5 Catalyst stability

The cracking experiments using Ni/RHC-4 catalyst were performed at 700 °C under microwave condition for ten cycles. Compared to the conversion efficiency for the first run, the conversion efficiency was only reduced by about 8% for the tenth run, indicating the catalyst was relatively stable for tar cracking under microwave condition.

**Fig. 7:**
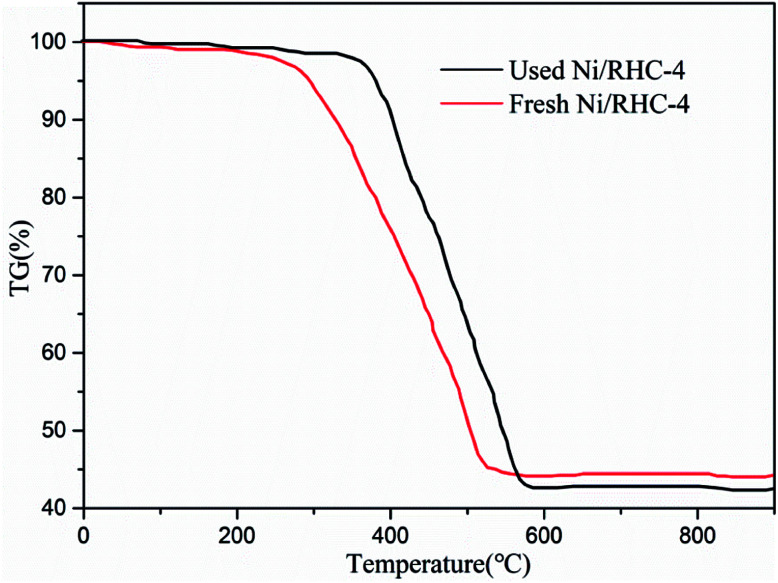
TG curves of fresh and used Ni/RHC-4 catalysts under air atmosphere.

For the fresh catalyst, a sharp weight loss was observed in the temperature range of about 300–520 °C in the TG curves (Fig. 7). In a comparison to the fresh catalyst, the temperatures at which the used catalyst experienced a rapid weight loss were only slightly increased to 350–580 °C. This suggested that the reactivity of the used catalyst was only slightly decreased, which can explain why the conversion efficiency was only reduced by about 8% for the tenth cycle. According to the TG analysis, only a little coke (about 2.7 wt% of the catalyst) was produced during tar cracking after the tenth cycle. Generally, nickel based catalysts used to tar removal are easily deactivated due to the generation of carbon deposition. For example, the amounts of the coke on Ni, Ni/Al_2_O_3_ and Ni/olivine catalysts used for tar removal could reach up to 8.3 wt%, 4.6 wt% and 9.06 wt%, respectively.^36–38^ This suggested that the Ni/RHC catalyst possessed a good resistance to coke deposition. This can be explained by the following two points: (1) part of the coke on the biochar can be consumed by steam during tar cracking and reforming; and (2) the presence of CO_2_, generated from the RHC pyrolysis and carboxyl group decomposition presented in the support, was also favorable to the consumption of the coke.^39^ The consumptions of the coke during tar cracking can in turn inhibit the deactivation of catalyst by producing new pores, particularly in the presence of the metallic species.^13^

## 4. Conclusion

Biomass pyrolysis tar cracking for the high quality syngas production over the Ni/RHC catalysts coupled with microwave heating was investigated. The biomass tar could be efficiently removed and the high quality syngas could be produced over the Ni/RHC catalyst under microwave condition. The temperature increase was favorable to the tar conversion and syngas production. The loading of nickel into the RHC could exert a significant positive effect on tar removal and syngas production. The tar conversion efficiencies were increased from 78.6% to 98.6% as the loading amount increased from 0 wt% to 16.86 wt%. When the loading amount was further increased from 10.42 wt% to 16.86 wt%, the tar conversion efficiency and syngas yield showed non-significant increases. The Ni/RHC catalysts became more effective on the tar removal and syngas production under microwave condition than under conventional condition. The Ni/RHC catalyst was relatively stable for tar cracking under microwave condition.

## Conflicts of interest

The authors declare that there is no conflict of interests.

## Supplementary Material
